# Supplementary material for: Programmable self-regulated molecular buffers for precise sustained drug delivery
Source: Nat Commun. 2022 Nov 2;13:6504. doi: 10.1038/s41467-022-33491-7 (PMC9630261; doi:10.1038/s41467-022-33491-7)
Supplement: Supplementary file 1 — Supplementary Information [file 41467_2022_33491_MOESM1_ESM.pdf]

Supplementary Information

Programmable, self-regulated molecular buffer for precise, sustained drug delivery

Author list

A. Desrosiers[1, 2], R.M. Derbali[3], S. Hassine[2], J. Berdugo[4], V. Long[5], D. Lauzon[1], V. De Guire[6], C. Fiset[5], L. DesGroseillers[2], J. Leblond Chain[3], A. Vallée-Bélisle[1, 2*]

Supplementary Text p. 2

Supplementary Figures (1 to 20) p. 7

Supplementary Tables (1 to 4) p. 27

Supplementary References p. 31

Supplementary Text

Binding curves: A concentration of doxorubicin or quinine smaller than the dissociation constant was used. The fluorescence values were corrected with a binding curve in the absence of doxorubicin or quinine and were then normalized (χ_{free}) with the maximum corrected fluorescence of doxorubicin or quinine. Binding curves were fitted the binding equation:

$$\chi_{free} = \frac{[aptamer]}{[aptamer]+K_D} \quad \text{(E1)}$$

The K_D values for all doxorubicin and quinine oligonucleotide are reported in Supplementary Table 2.

Approach to vary the K_D of the buffers: For quinine, our hypothesis was that site specific mutations could likely reduce the binding affinity for quinine (this strategy would likely not lead to an increase in binding affinity). We have explored various mutations (20) that were based on the three-way junction Q0 DNA aptamer. We hypothesized that quinine would bind in the three-way junction and thus modification of the stems (either stabilizing, destabilizing, increasing length, decreasing length) while maintaining the three-way junction secondary structure would yield similar or lower binding affinity for quinine. With this strategy, we had a success rate of around 30% (lots of sequence were either of similar K_D as previously obtained, unchanged K_D or K_D that were too high). Of note, this strategy remains cumbersome in absence of structural information on the aptamer and its binding site since the mutations will remain random.

For doxorubicin, we did not alter the D0 sequence since doxorubicin binding affinity for DNA duplex with GC bp doesn't vary. To circumvent this limitation, we hypothesized that G-quadruplex sequences would also bind doxorubicin since there a multiple G-G stacking which was indeed the case. The affinity of doxorubicin was also different with different G-quad sequence (they all have different structure and thus we can work with different K_D). We used sequences available in the literature.

Stoichiometry curves: Binding affinities were corrected to take into account the binding stoichiometry (e.g. some G-quadruplexes form a dimer that binds 1 doxorubicin, which gives a stoichiometry of 0.5 per ssDNA). To measure stoichiometry, binding curves at doxorubicin or quinine concentrations above the K_D (i.e. ligand depletion regimen) were performed. A ligand depletion regimen gives a linear decrease of fluorescence upon DNA aptamer addition and a plateau when saturation is reached (e.g. Supplementary Fig. 3C). The linear decrease and the plateau were each fitted with a linear regression:

$$Y(X) = aX + b \quad \text{(E2)}$$

The stoichiometry *n* corresponds to the initial drug concentration (doxorubicin or quinine) divided by the abscissa intercept of the two linear fit:

$$n = \frac{D_0}{\left(\frac{b_2 - b_1}{a_1 - a_2}\right)} \quad \text{(E3)}$$

The error on stoichiometry was determined with the 95% confidence interval of the abscissa intercept (*1*). Binding curves were then corrected with the stoichiometry:

$$\chi_{free} = \frac{n \cdot [DNA]}{n \cdot [DNA] + K_{\mathrm{D}}} \quad \text{(E4)}$$

The stoichiometries for all doxorubicin and quinine oligonucleotides are reported in Supplementary Table 2.

<u>Buffer titration curves:</u> To characterize the buffer zones of molecular buffers, buffer titrations such as acid-base titration were performed. Typically, acid or base is added to a buffer solution, and its pH is measured with each acid-base increment. Here, acid and base are replaced by doxorubicin or quinine (drug), and the free drug concentration is measured (pH= log [H^+]). Free drug concentration was obtained by recording fluorescence values for every drug increment, which were then converted with a fluorescence standard curve. Free drug concentration is considered active, and the bound drug is considered inactive. The free drug concentration was plotted against the total added drug amounts, which gives a typical biphasic curve similar to acid-base buffer titrations (where pH is plotted against total acid/base added). For example, as with acid-base titration, the buffer zone is the plateau before equivalence (i.e. the zone where the drug increment does not significantly change the free drug concentration). The binding equation was used to fit the data:

$$[D]_{free} = \frac{D_0 - T_0 - K_{\mathrm{D}} + \sqrt{(T_0 - D_0 + K_{\mathrm{D}})^2 + 4(D_0 \cdot K_{\mathrm{D}})}}{2} \quad \text{(E5)}$$

To quantitatively measure the buffer zone, the same definition for buffer capacity as used for acid-base buffers was used: the buffer capacity is the infinitesimal change of pH upon addition of an infinitesimal amount of acid or base:

$$\beta = \frac{dn}{d(pH)} \quad \text{(E6)}$$

Here, buffer capacity would be the infinitesimal change of free drug concentration upon the addition of an infinitesimal amount of drug. The buffer capacity values were calculated according to equation E5 for every drug increment. For acid-base titration, buffer capacity peaks when pH=pKa or here when [free drug]= K_{D}. To verify this, buffer capacity values obtained from buffer titration curves were plotted against their corresponding free drug concentration and were fitted with the derivative of equation E5:

$$\beta = 2.303 \cdot [D]_{free} \frac{\left([D]_{free} + K_{\mathrm{D}}\right)^2 + T_0 K_{\mathrm{D}}}{\left([D]_{free} + K_{\mathrm{D}}\right)^2} \quad \text{(E7)}$$

Molecular buffer programming: To build self-regulated DDS, it is important to rationally program the free drug concentration released by the buffer. It is possible to program a desired free drug concentration by modifying the [Drug]/[Buffer] ratio (e.g. when the ratio is approximately 0.5, the free drug concentration is equal to the K_D):

$$i = \frac{D_0}{T_0} \tag{E8}$$

In this manuscript, 10 µM of 0.5-D0 refers to 10 µM of doxorubicin and 20 µM of D0 DNA buffer.

To rationally determine the ideal [Drug]/[Buffer] ratio, the desired free drug concentration $[D]_{free}$ is used in the equation to predict the ratio:

$$i = \frac{[D]_{free}([D]_{free}+T_0+K_D)}{T_0([D]_{free}+K_D)} \tag{E9}$$

Here, the molecular buffer concentration is chosen based on the need for a large drug reservoir or a smaller one. Increasing buffer concentration and keeping the same [Drug]/[Buffer] ratio (eq. E9) allows an increase in the amount of drug that is sequestered in the buffer, like a buffer reservoir, while keeping the same free drug concentration. For molecular buffers to act as buffer reservoirs, their concentration needs to be higher than the K_D to ensure binding does not depend on it. Therefore, buffer reservoir size is defined by the ratio:

$$R = \frac{T_0}{K_D} \tag{E10}$$

Dilution curves: The buffer effect of molecular buffers for DDS was characterized by dilution experiments to simulate both drug (doxorubicin) and buffer degradation. To do so, the buffer reservoir size was increased from to 0 to 1600x according to equation E10. The total doxorubicin concentration needed to achieve a free doxorubicin concentration equal to the K_D was determined with equation E9. Every hour, 1/2 dilution was performed, and the resulting free doxorubicin concentration was measured. The dilution experiments were fitted with a binding equation with both doxorubicin and buffer degradation decay:

$$[D]_{free} = \frac{\left((D_0 \cdot e^{(-\lambda t)}+c)-(T_0 \cdot e^{(-\lambda t)}+d)-K_D\right)+\sqrt{\left((T_0 \cdot e^{(-\lambda t)}+d)-(D_0 \cdot e^{(-\lambda t)}+c)+K_D\right)^2+4\left((D_0 \cdot e^{(-\lambda t)}+c) \cdot K_D\right)}}{2} \tag{E11}$$

The buffer reaches equilibrium with doxorubicin in milliseconds (see section on binding kinetics) and is therefore always in pre-equilibrium with the dilution rate. This allows the use of the equilibrium-based equation E11 to fit dilution experiments. Equation E11 gave a fitting for the K_D and the half-lives of doxorubicin and buffer. For the control with no buffer, an exponential decay fit was used:

$$D(t) = D_0 \cdot e^{(-\lambda t)} + c \tag{E12}$$

The free doxorubicin half-life was calculated for each dilution curve with a logistic function:

$$[D]_{free}(t) = \frac{[D]_{t_0}}{1+e^{k\left(t-t_{\frac{1}{2}}\right)}} \tag{E13}$$

The half-life obtained from the dilution data was plotted against the buffer reservoir size R ([Drug]/[Buffer] ratio is defined with equation E9 since it varies with the buffer reservoir size):

$$t_{\frac{1}{2}} = \left(\frac{\ln\left(\left([D]_{t_{\frac{1}{2}}}^{2} + [D]_{t_{\frac{1}{2}}} \right) \cdot K_{\mathrm{D}} \right)}{\left(R \cdot K_{\mathrm{D}} \left(i \cdot [D]_{t_{\frac{1}{2}}} - [D]_{t_{\frac{1}{2}}} + i \cdot K_{\mathrm{D}} \right) \right)} \right) \cdot \frac{-1}{\lambda} \tag{E14}$$

DNAse assays: Molecular buffer degradation rate was programmed by adding increasing amounts of DNAse I (New England Biolabs, Ipswich MA). Doxorubicin fluorescence was measured over time and converted to total DNA aptamer concentration:

$$T_0 = D_0 - [D]_{free} - K_{\mathrm{D}} + \frac{D_0 \cdot K_{\mathrm{D}}}{[D]_{free}} \tag{E15}$$

Degradation of DNA over time by DNAse I was fitted with a double exponential decay:

$$T(t) = T_a \cdot \mathrm{e}^{(-\lambda_a t)} + T_b \cdot \mathrm{e}^{(-\lambda_b t)} + c \tag{E16}$$

Equation E16 allowed fitting for doxorubicin release caused by DNA aptamer degradation:

$$[D]_{free} = \frac{\left(D_0 - \left(T_a \cdot \mathrm{e}^{(-\lambda_a t)} + T_b \cdot \mathrm{e}^{(-\lambda_b t)} + c \right) - K_{\mathrm{D}} \right) + \sqrt{\left(\left(T_a \cdot \mathrm{e}^{(-\lambda_a t)} + T_b \cdot \mathrm{e}^{(-\lambda_b t)} + c \right) - D_0 + K_{\mathrm{D}} \right)^2 + 4(D_0 \cdot K_{\mathrm{D}})}}{2} \tag{E17}$$

Dialysis kinetics: The impact of the molecular buffer degradation rate on its transported drug (doxorubicin) was investigated with a dialysis setup. In this setup, doxorubicin flowed out of the dialysis cassette, and the fluorescence inside the cassette was measured. Therefore, doxorubicin dialysis simulated its degradation, and addition of DNAse I in the cassette increased the molecular buffer decay. Fluorescence values were converted to free doxorubicin concentration as described previously and plotted against time:

$$[D]_{free} = \frac{\left(\left(D_0 \cdot \mathrm{e}^{(-\lambda t)} + d \right) - \left(T_a \cdot \mathrm{e}^{(-\lambda_a t)} + T_b \cdot \mathrm{e}^{(-\lambda_b t)} + c \right) - K_{\mathrm{D}} \right) + \sqrt{\left(\left(T_a \cdot \mathrm{e}^{(-\lambda_a t)} + T_b \cdot \mathrm{e}^{(-\lambda_b t)} + c \right) - \left(D_0 \cdot \mathrm{e}^{(-\lambda t)} + d \right) + K_{\mathrm{D}} \right)^2 + 4\left(\left(D_0 \cdot \mathrm{e}^{(-\lambda t)} + d \right) \cdot K_{\mathrm{D}} \right)}}{2} \tag{E18}$$

DNA degradation in mouse serum: Buffer degradation rate was measured in whole mouse serum, and various backbones were tested to vary the degradation rate (instead of employing DNAse I). SYBR green was used in excess to monitor DNA aptamer degradation in serum with fluorescence through time. The degradation rate was determined with a single exponential decay:

$$F(t) = F_0 \cdot \mathrm{e}^{(-\lambda t)} + b \tag{E19}$$

<u>HPLC quantification of doxorubicin:</u> Doxorubicin underwent degradation in mouse serum, and various DNA aptamers were used as molecular buffers for doxorubicin. The impact of DNA aptamers with various decay kinetics was assessed by measuring total doxorubicin degradation in mouse serum. To do so, doxorubicin degradation was fitted with a single exponential decay, as in equation E19. D0-thioate was fitted with a double exponential decay:

$$D(t) = D_a \cdot e^{(-\lambda_a t)} + D_b \cdot e^{(-\lambda_b t)} + b \tag{E20}$$

<u>Toxicity assay:</u> Here, the molecular buffer concentration is programmed to deliver a precise free doxorubicin concentration in the presence of a large reservoir of inactive drug. To confirm the concentration of free doxorubicin, a toxicity assay with HeLa cells was performed. In this assay, we increased doxorubicin concentrations, tested for cell viability, and measured IC_{50} values. We then programmed different drug/buffer formulations using equation E9 that deliver different specific free doxorubicin concentrations. If the molecular buffer delivers the same free doxorubicin concentration, it should have the same toxicity. Toxicity (IC_{50}) assays were fitted with a 4-parameter logistic fitting:

$$Viability = \frac{(min - max)}{1 + \left(\frac{[Doxo]}{IC50}\right)^k} + max \tag{E21}$$

The capacity of molecular buffers to deliver a precise doxorubicin concentration was also validated by increasing the buffer reservoir size while maintaining the same free doxorubicin concentration. This allowed showing that it is possible to use a large reservoir of inactive drug.

<u>Error propagation of a ratio:</u> To calculate the standard deviation of a ratio, the formula for error propagation is different (*2*). For a ratio z=x/y, the error propagation is:

$$\Delta z = z \cdot \sqrt{\left(\frac{\Delta x}{x}\right)^2 + \left(\frac{\Delta y}{y}\right)^2} \tag{E22}$$

Supplementary Figures

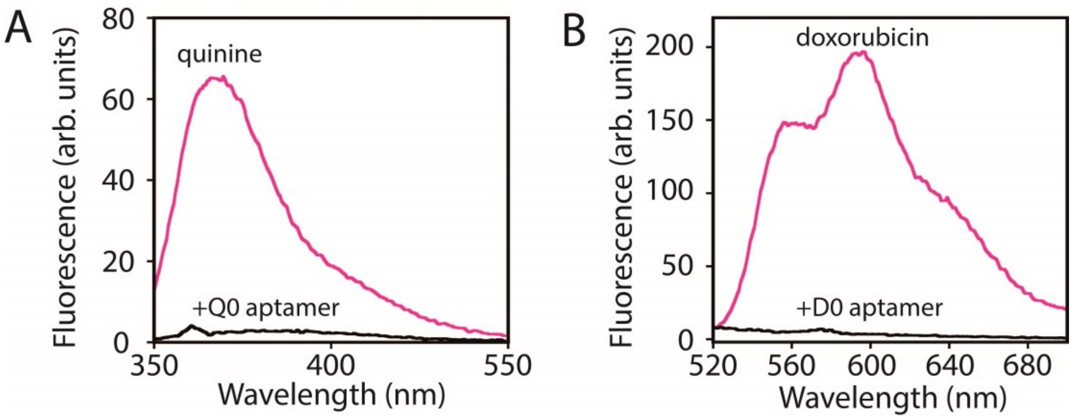

Supplementary Fig. 1. Fluorescence emission spectra of 1 μM quinine (A) and 1 μM doxorubicin (B) with (black line) or without (pink line) 25 μM of their specific DNA-binding aptamer.

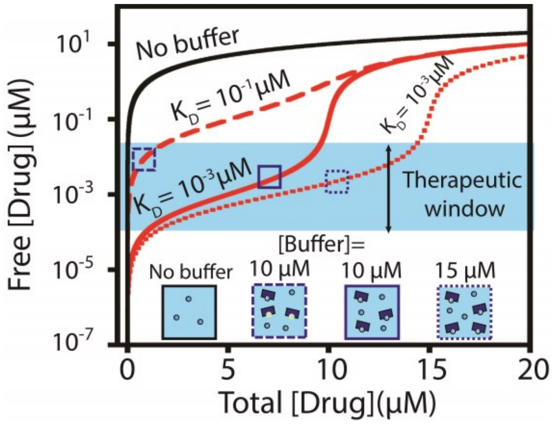

Supplementary Fig. 2. The buffer capacity of a buffer can be readily determined using a titration curve (i.e. like a proton titration for acid-base buffers), which displays a "buffer zone" near the K_D (i.e. pK_A), in which the free drug concentration remains relatively constant upon increasing the total drug concentration. Increasing the K_D enables an increase in the free concentration of the drug (red line vs red dashed line), while increasing the buffer concentration extends the buffer capacity to higher drug concentrations (red line vs red dotted line). Drug increase in absence of buffer is depicted by the black line (y axis is a log scale).

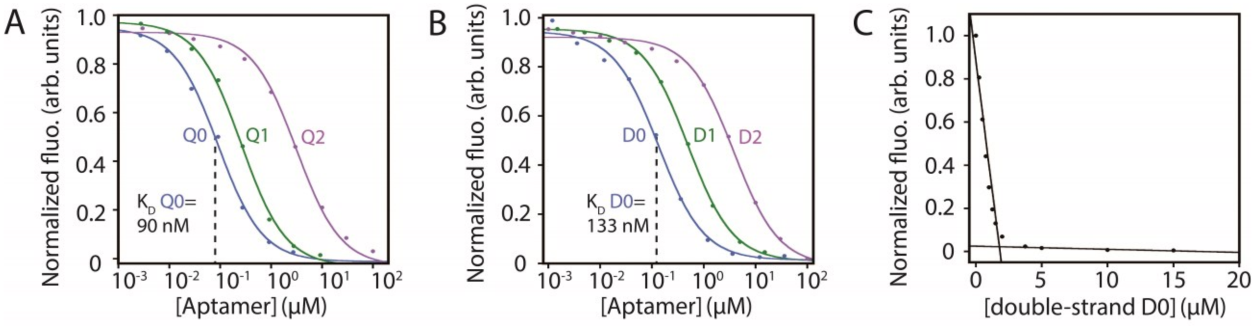

Supplementary Fig. 3. Binding curves of the: A) quinine-binding aptamer variants (Q0=blue line, Q1= green line, Q2= purple line); and B) doxorubicin-binding aptamers (D0= blue line, D1= green line, D2= purple line). See Supplementary Table 1 for DNA buffer sequences. Data were fitted with equation E1 (see Supplementary Text). (C) Binding in ligand depletion regimen of D0 with doxorubicin to measure binding stoichiometry. In this experiment, 5 µM of doxorubicin was completely bound by 2.5 µM of D0-buffer, which gives a stoichiometry of 2.72 ± 0.4 doxo per double-stranded D0-buffer (intercept, see Supplementary Table 1). Data were fitted with equation E2 and E3 (see Supplementary Text).

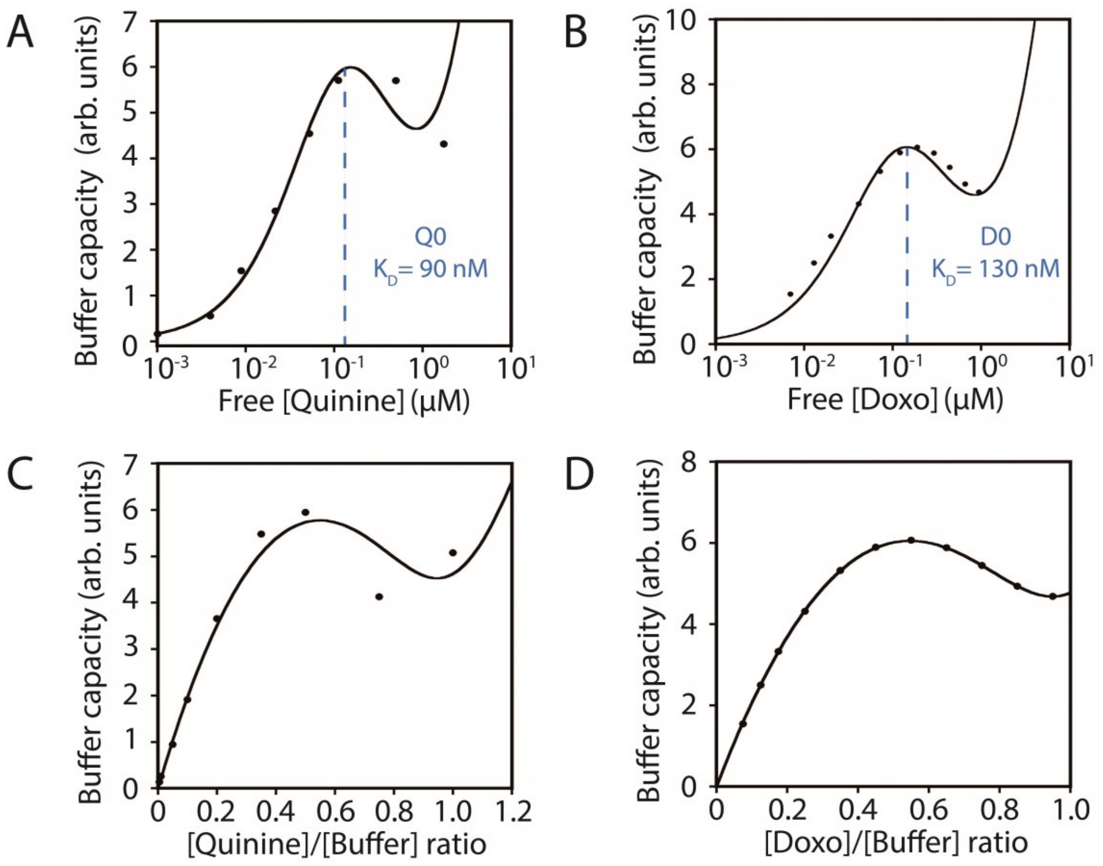

Supplementary Fig. 4. (A, B) The highest molecular buffer capacity is reached at a free drug concentration equal to the K_D (e.g. maximum buffer capacity for acid-base buffer is reached at a pH = pK_A). Data were fitted with equation E7 (see Supplementary Text). The optimal buffer capacities (β) were found at 135 ± 21 nM ($\beta^{max}_{quinine}$) and 128 ± 7 nM (β^{max}_{doxo}). See Supplementary Table 1 for buffer DNA sequences. (C, D) A drug/buffer concentration ratio of 0.5 yields its highest buffer capacity value with a free drug concentration equal to the K_D. Data were fitted with equation E7 (see Supplementary Text).

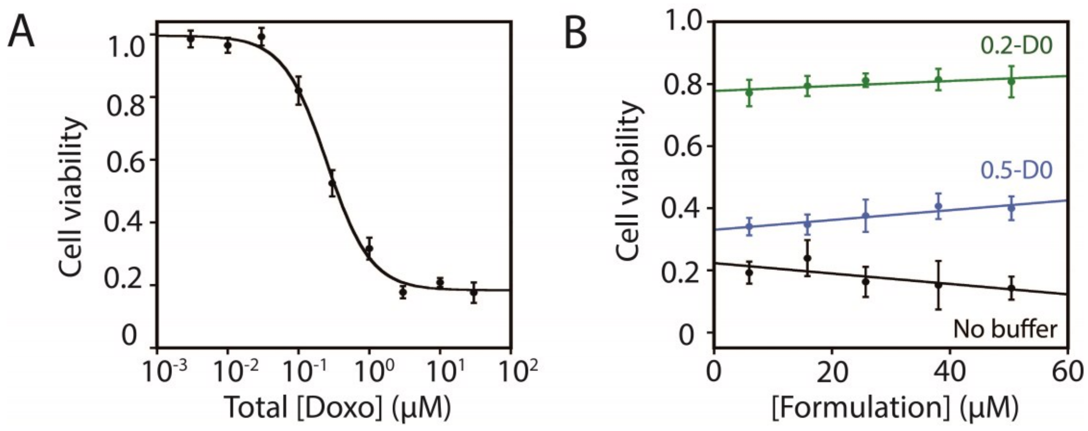

Supplementary Fig. 5. (A) Cytotoxicity of doxorubicin on HCT116 cells assayed with resazurin (n=8 biologically independent samples per data point). Data were fitted with equation E21 (see Supplementary Text). (B) HCT116 cells are only sensitive to free doxorubicin: the doxo-buffer formulation is not toxic even at a high concentration, see for example formulation 0.2-D0 (n=8 biologically independent samples per data point). Black line: without molecular buffer, blue line: formulation 0.5-D0, green line: formulation 0.8-D0 (a formulation is defined as the drug/buffer concentration ratio, as 0.5-D0 corresponds to a 2x excess of buffer vs drug concentration). For both panels, data are presented as mean values +/- SD.

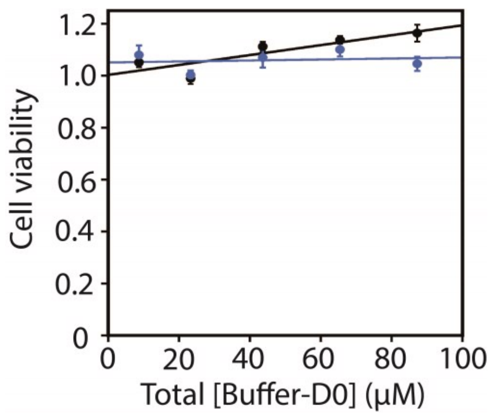

Supplementary Fig. 6. HeLa (blue line) and HCT116 (black line) cell lines viability in the presence of increasing amounts of D0 buffer (n=8 biologically independent samples per data point). Data are presented as mean values +/- SD.

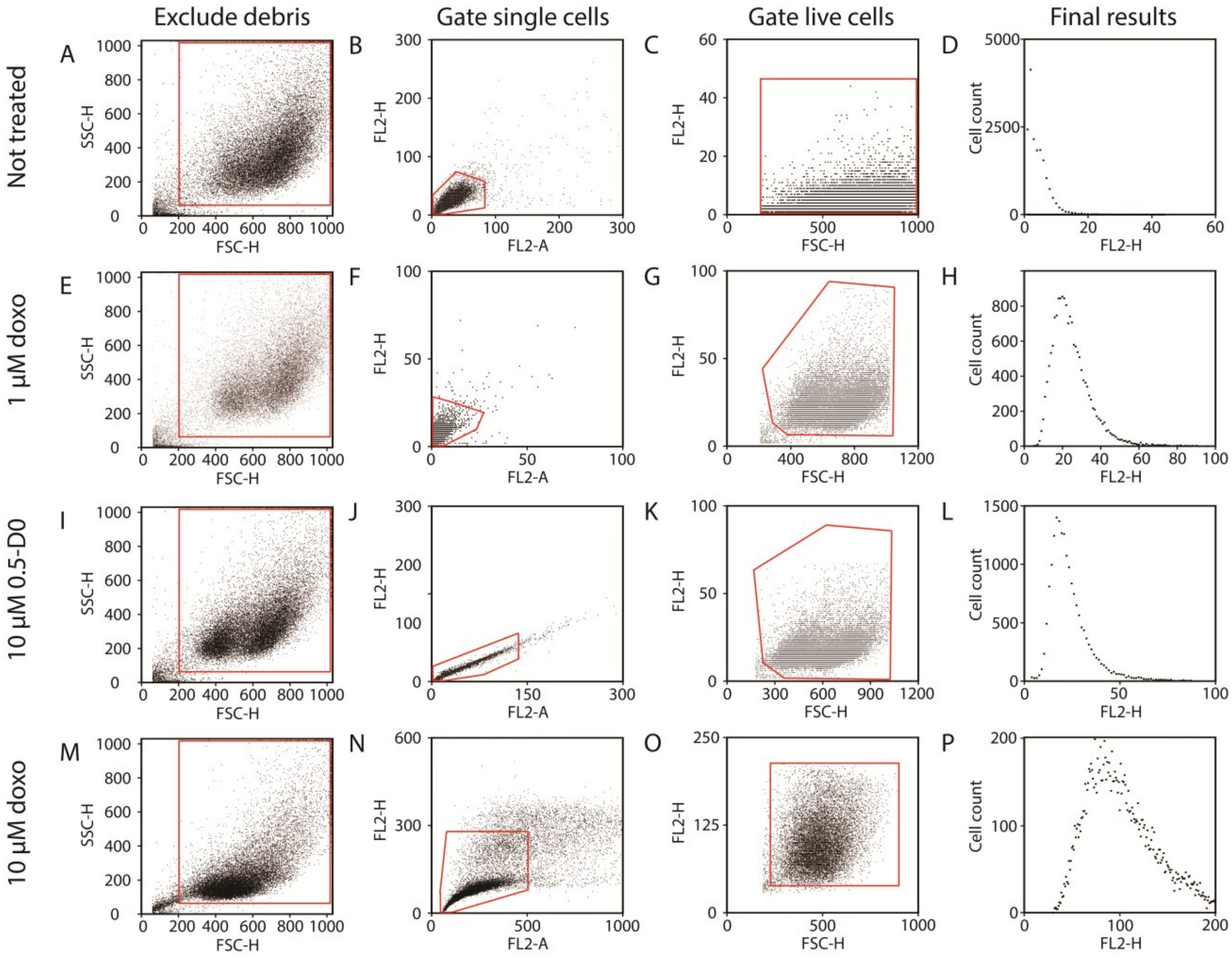

Supplementary Fig. 7. Gating strategy for FACS experiments on HeLa cells for all groups. First cell debris were excluded (A, E, I and M). Doublets signal was thene excluded (B. F, J and N). Cells with very low doxorubicin signal were excluded in the live cell selection (C, G, K and O) which allowed for the final histogram plotting of doxorubicin signal with cell count (D, H, L and P). Every row corresponds to a complete gating strategy for a specific group: not treated (A-D), 1 μM free doxorubicin (E-H), 10 μM drug buffer formulation (I-L) and 10 μM doxorubicin representing only the drug reservoir (M-P).

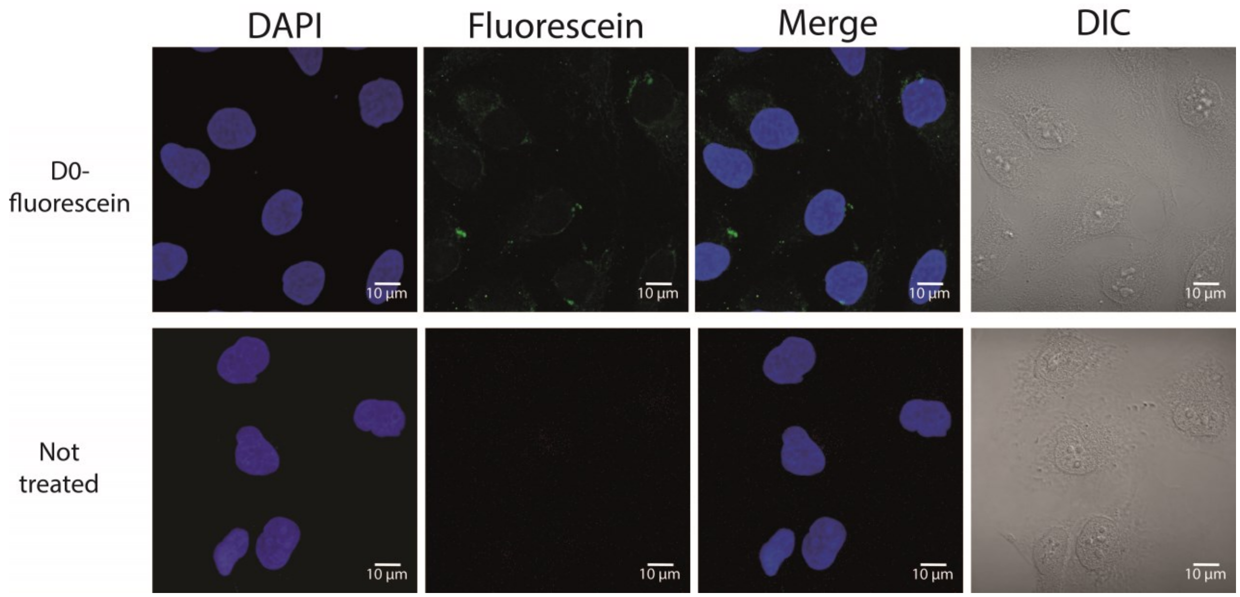

Supplementary Fig. 8. Cellular uptake of D0-fluorescein molecular buffer displays cytoplasmic localization. DAPI is employed to stain the nuclei. For each condition, n= 3 independent experiments.

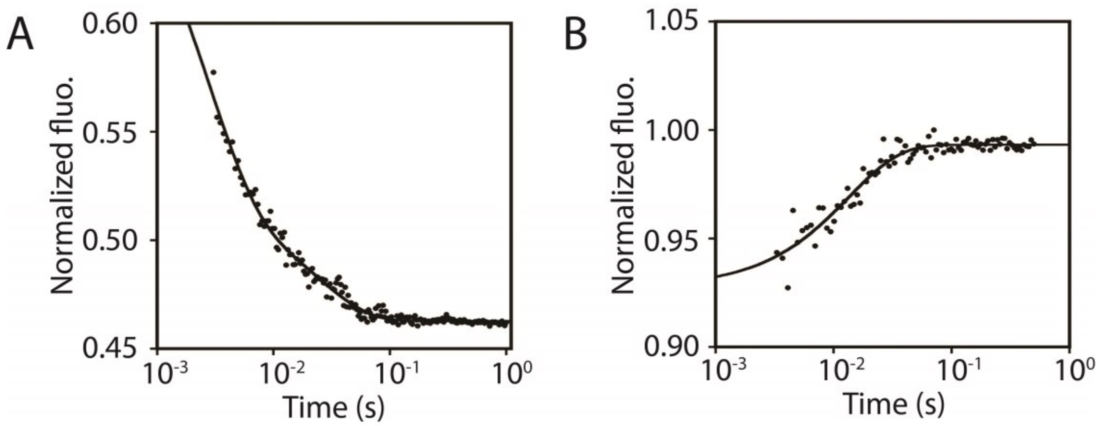

Supplementary Fig. 9. Association and dissociation kinetics of doxorubicin with the D0-buffer. A) Association kinetics were monitored following rapid mixing of the D0-buffer to a doxorubicin solution using a stopped-flow fluorimeter (data were fitted with a double exponential function). (B) Dissociation kinetics were monitored following a rapid 6-fold dilution (1/6) of a doxorubicin/D0-buffer solution (data were fitted with a single exponential). Of note: for both experiments (association and dissociation), the kinetic was faster than the stopped-flow fluorimeter dead-time ~3 ms, meaning only partial data of the kinetic was acquired.

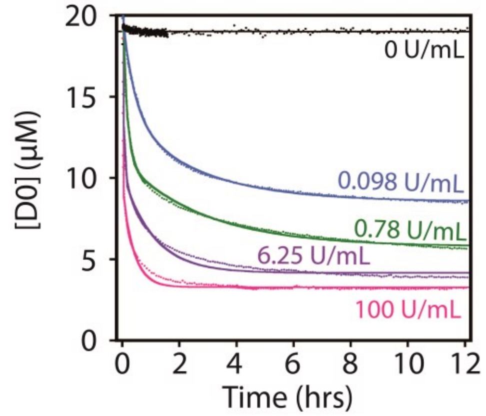

Supplementary Fig. 10. Degradation kinetics of 20 μM D0 molecular buffer by DNAse I (data were fitted with equation E16- see Supplementary Text). Buffer concentration was determined by using doxorubicin fluorescence (as the buffer gets degraded, more doxorubicin is released). Black line: without DNAse I; blue line: 0.098 U/mL of DNAse I; green line: 0.78 U/mL of DNAse I; purple line: 6.25 U/mL of DNAse I and pink line: 100 U/mL of DNAse I.

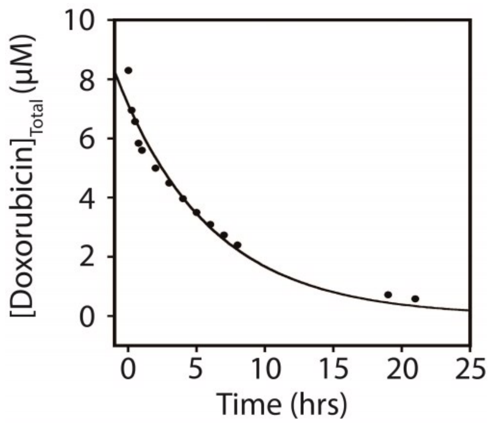

Supplementary Fig. 11. Dialysis kinetics of doxorubicin (9.3 μM) from a dialysis cassette with a 3-kDa molecular weight cut-off (MWCO). Data were fitted with a single exponential function.

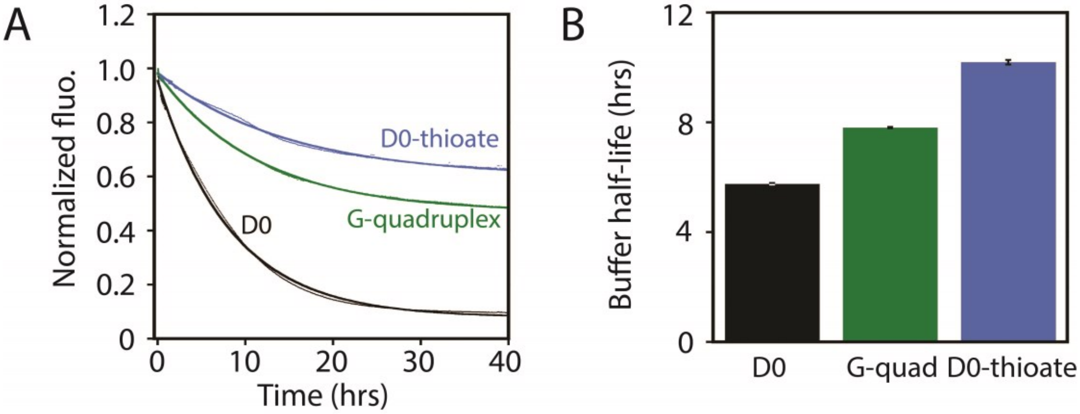

Supplementary Fig. 12. (A) Buffer degradation kinetics in whole mouse serum (data were fitted with a single exponential function). (B) Buffer D0-thioate and G-quadruplex variants display enhanced chemical stability in whole mouse serum (half-life). Black line/bar: D0 buffer, green line/bar: G-quad buffer, blue line/bar: D0-thiate buffer. Data are presented as mean values and error bars are derived from a single fitting of the kinetic from panel A (and thus not derived from multiple replicates). Buffer concentrations were measured by taking advantage of SYBR green-enhanced fluorescence upon binding to duplex or quadruplex DNA structures.

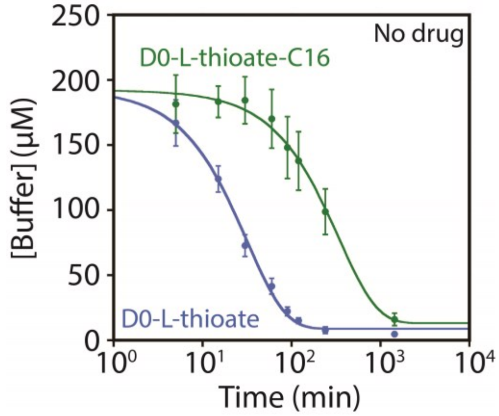

Supplementary Fig. 13. Pharmacokinetics of D0-L-thioate (blue line) and D0-L-thioate-C16 (green line) buffer in CD-1 female mice (n=6, 55 mg/kg IV injection). Data were fitted with equation E18 (see Supplementary Text). Data are presented as mean values +/- SD.

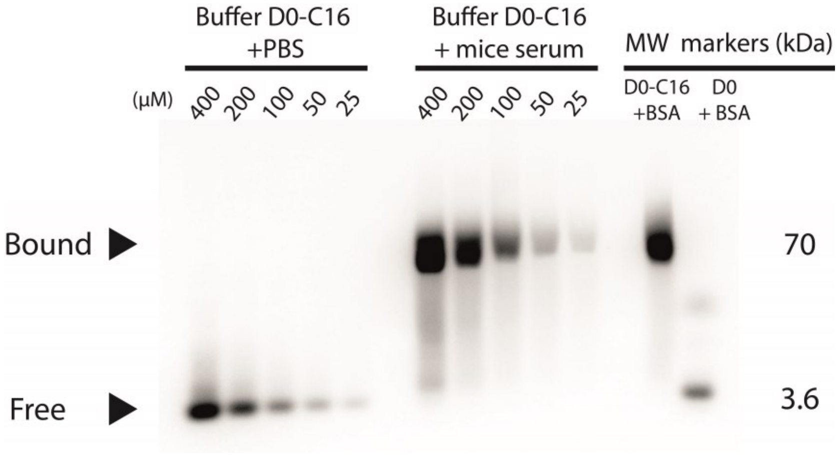

Supplementary Fig. 14. Agarose electrophoresis gel demonstrating the capacity of albumin to bind high concentrations of our D0-C16 buffer. The bound complex in mouse serum has the same R_f as the albumin-buffer complex from MW markers. In the absence of C16 fatty acid, D0 does not bind to albumin. All oligonucleotides were labeled with fluorescein for gel imaging. Samples were prepared by adding the corresponding amount of fluorescent DNA in either 50% glycerol/PBS, 50% glycerol/mouse serum or 50% glycerol/500 µM albumin solution in PBS. MW markers: 70 kDa (D0-C16 bound to albumin) and 3.6 kDa (D0 only since it cannot bind albumin). This experiment was repeated 3 independent times.

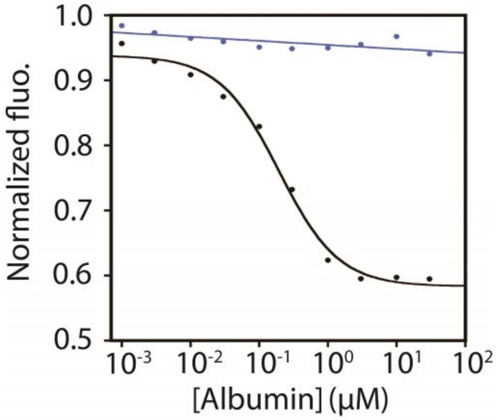

Supplementary Fig. 15. Binding curve of D0-L-thioate-C16 (black line) and D0-L-thioate (blue blue) buffer with bovine serum albumin. D0-thioate-C16 buffer were labeled with fluorescein in close proximity to the C16 fatty acid moiety to generate fluorescence quenching upon binding to albumin. Fitted K_D was 193 ± 30 nM (data were fitted with equation E1 for the albumin binding oligonucleotide- see Supplementary Text).

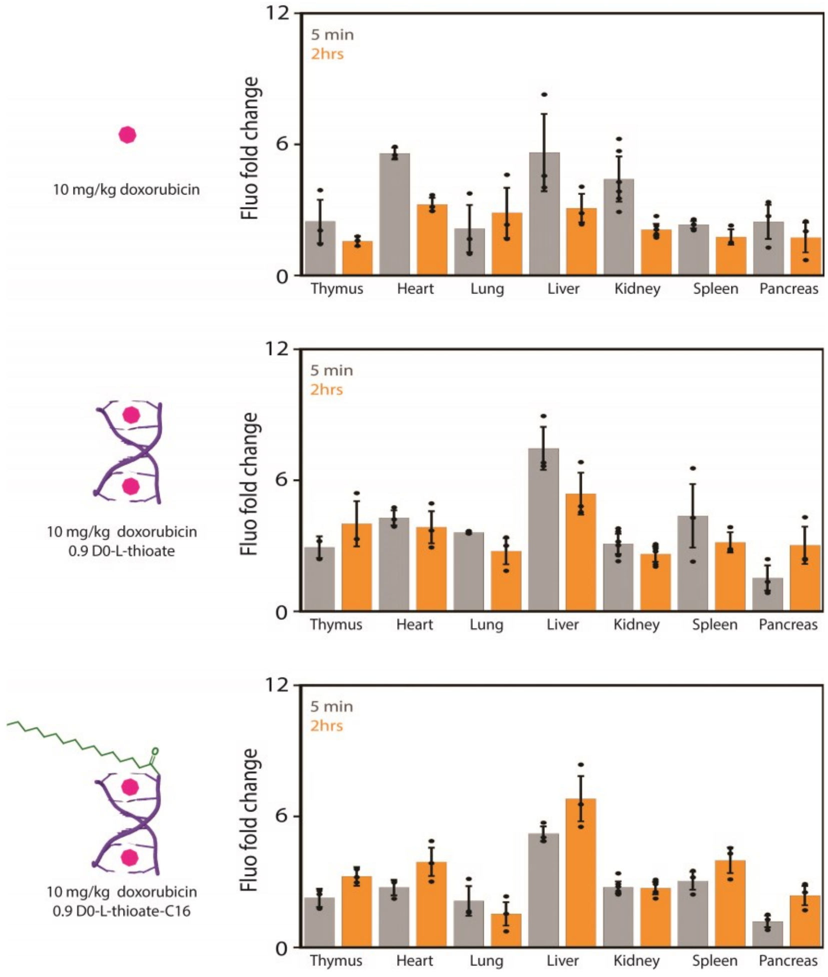

Supplementary Fig. 16. Doxorubicin fluorescence levels detected in various organs 5 min (gray bars) or 2 hrs (orange bars) after IV injection with various doxorubicin formulations (n=3 for each organ and time point). Top: 10 mg/kg doxorubicin; Middle: 0.9-D0-L-thioate formulation; and Bottom: 0.9-D0-L-thioate-C16 formulation. Fluorescence variations were normalized with saline-injected mice. Doxorubicin fluorescence levels in the organs were determined using an IVIS spectrum instrument (see Methods section). Data are presented as mean values of fold change +/- SD.

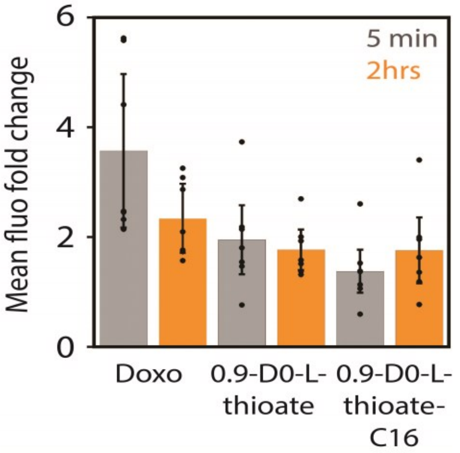

Supplementary Fig. 17. Mean doxorubicin fluorescence fold changes from the seven organs of Supplementary Fig. 15 at 5 min (gray bars) or 2 hrs (orange bars) after IV injection with: doxorubicin alone, 0.9-D0-L-thioate formulation 0.9-D0-L-thioate-C16 formulation. For all individual organs and time point, n=3. Fluorescence variations were normalized with saline-injected mice. Doxorubicin fluorescence levels in the organs were determined using an IVIS spectrum instrument (see Methods section). Data are presented as mean values of fold change +/- SD.

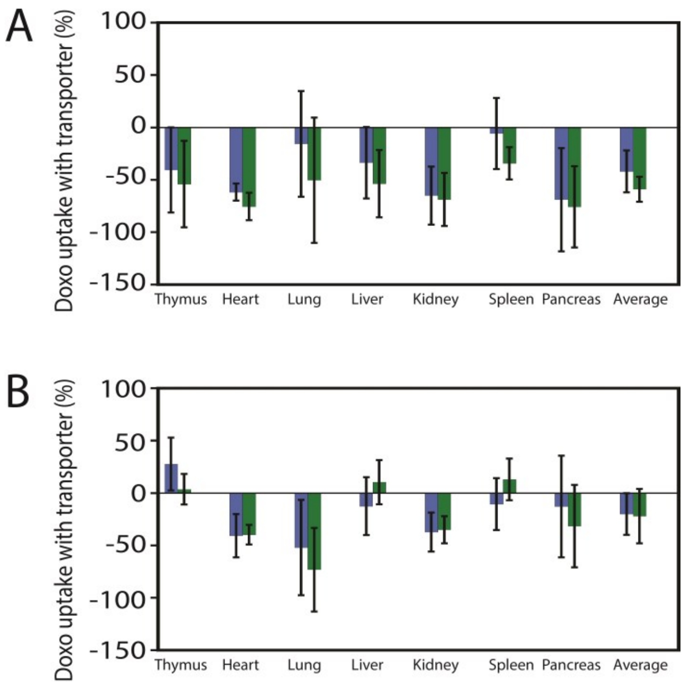

Supplementary Fig. 18. *Ex vivo* doxorubicin fluorescence fold changes in mouse organs with doxo/buffer formulations versus doxorubicin alone (A: 5 min; B: 2 hrs). Each bar represents a single ratio of fluorescence from either formulations divided by doxorubicin alone (z=formulation/doxo). Blue bars: 0.9-D0-L-thioate formulation (10 mg/kg doxorubicin, 55 mg/kg D0-L-thioate); Green bars: 0.9-D0-L-thioate-C16 formulation (10 mg/kg doxorubicin, 55 mg/kg D0-L-thioate-C16). For all conditions, n=3 (each bar is a ratio of n=3/n=3, meaning a total of n=6 for the ratio). Doxorubicin fluorescence levels were determined using an IVIS spectrum instrument (see Methods section). Data are presented as mean values of fold change and all error bars are calculated with error propagation formula for ratios (see equation E22).

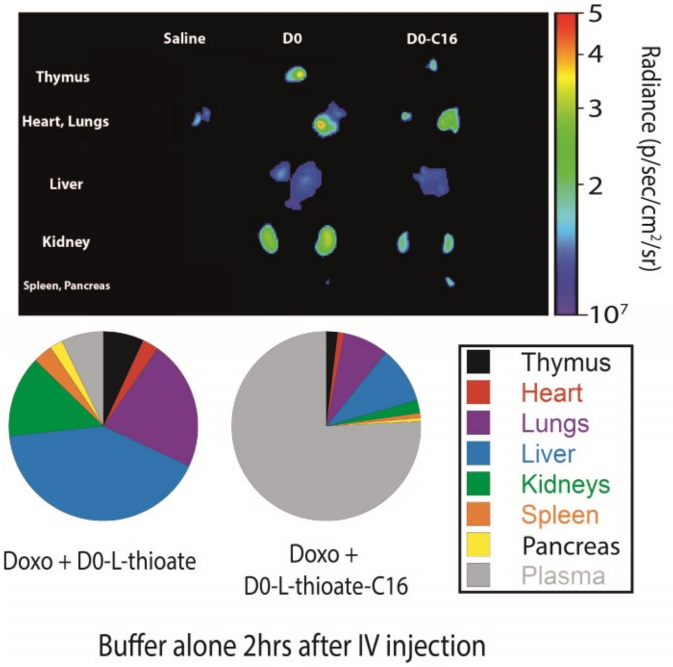

Supplementary Fig. 19. Top: *Ex vivo* fluorescence imaging of DNA buffers in mouse organs 2 hrs after injection without doxorubicin (n=3, 55 mg/kg). Bottom: DNA buffer distributions 2 hrs after injection (the buffer concentration in plasma was determined using HPLC measurements).

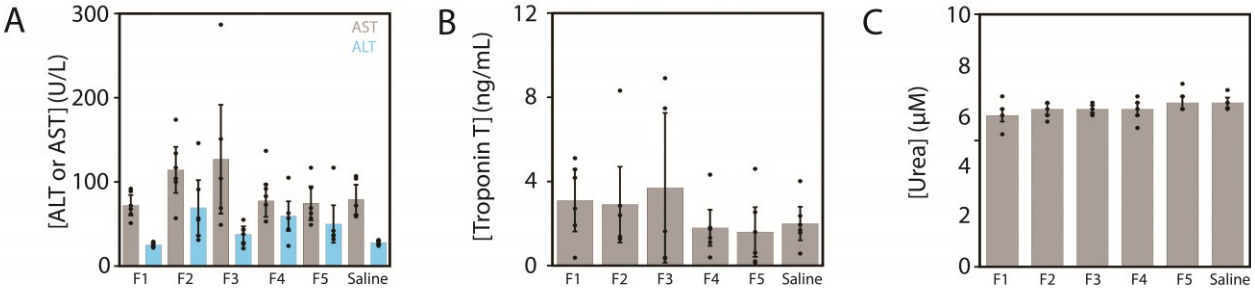

Supplementary Fig. 20. Eight-week-old CD-1 female mice were injected with various doxorubicin formulations, and various biomarkers were measured two weeks after injection to assess toxicity: (A) liver enzymes (AST: light gray bars, ALT: dark gray bars); (B) Troponin T; and (C) blood urea. F1: doxorubicin alone (10 mg/kg doxorubicin); F2: 0.9-D0-L-thioate formulation (10 mg/kg doxorubicin, 55 mg/kg D0-L-thioate); F3: 0.9-D0-L-thioate-C16 formulation (10 mg/kg doxorubicin, 55 mg/kg D0-L-thioate-C16); F4: D0-L-thioate alone (55 mg/kg D0-L-thioate); F5: D0-L-thioate-C16 alone (55 mg/kg D0-L-thioate-C16). For all conditions, n=6. For each group, n=6 and data are presented as mean values +/- SD.

Supplementary Tables

Supplementary Table 1. Oligonucleotides sequences for doxorubicin and quinine.

Name	Sequence
D0	5′─ CGC GCG CGC GCG ─3′
D1	5′─ GGG CTT TTG GGC ─3′
D2	5′─ GGT TGG TGT GGT TGG ─3′
G-quad	5′– GGG CTT TTG GGC–3′
D0-FAM	5′─ CGC GCG CGC GCG ─(fluorescein C3)─3′
D0-C16	5′─ CGC GCG CGC GCG ─(C16)─3′
D0-chol	5′─ (cholesterol) ─ CGC GCG CGC GCG ─3′
D0-PS	5′─ C*G*C* G*C*G* C*G*C* G*C*G ─3′ *phosphorothioate linkage
D0-L-thioate	5′─ C*G*C* G*C*G* C*G*C* G*C*G* C*G*C* G*C*G* C*G*C* G*C*G ─3′<br>*phosphorothioate linkage
D0-L-thioate-FAM	5′─ C*G*C* G*C*G* C*G*C* G*C*G* C*G*C* G*C*G* C*G*C* G*C*G ─(fluorescein C3)─3′<br>*phosphorothioate linkage
D0-L-thioate-C16	5′─ C*G*C* G*C*G* C*G*C* G*C*G* C*G*C* G*C*G* C*G*C* G*C*G ─(C16)─3′<br>*phosphorothioate linkage
D0-L-thioate-FAM-C16	5′─ C*G*C* G*C*G* C*G*C* G*C*G* C*G*C* G*C*G* C*G*C* G*C*G ─(fluorescein C3)─(C16)─3′<br>*phosphorothioate linkage
Q0	5′─ GGC GAC AAG GAA AAT CCT TCA ACG AAG TGG GTC GCC ─3′
Q1	5′─ GAC AAG GAA AAT CCT TCA ACG AAG TGG GTC G ─3′
Q2	5′─ GGC GAC AAG GAA AAT CCT TTC ACG AAG TGG GTC GCC G ─3′

Supplementary Table 2. Binding affinities and stoichiometry of DNA buffers for doxorubicin and quinine in PBS or biological solutions.

Sequence	K_D (µM)	ΔG (kcal·mol^{-1})	Stoichiometry per ssDNA	Medium	Temperature (°C)
D0	0.133 ± 0.02	-9.76 ± 0.07	1.36 ± 0.2	Phosphate sodium	20
D1	0.703 ± 0.08	-8.73 ± 0.06	0.536 ± 0.04		
D2	3.76 ± 0.3	-7.70 ± 0.05	1.01 ± 0.07		
D0	0.510 ± 0.03	-8.93 ± 0.1	1.21 ± 0.1	DNAse buffer	37
D0	1.06 ± 0.04	-8.48 ± 0.3	1.29 ± 0.06	Mouse serum	37
D0-thioate	1.02 ± 0.02	-8.50 ± 0.2	1.35 ± 0.06		
G-quad	3.06 ± 0.7	-7,83 ± 0.01	1.21 ± 0.1		
D0-L-thioate	0.592 ± 0.1	-8,838 ± 0.1	2.75 ± 0.3		
D0-L-thioate-C16	0.578 ± 0.1	-8,853 ± 0.1	2.74 ± 0.3		
D0	0.968 ± 0.1	-8.53 ± 0.06	1.29 ± 0.2	Cell culture medium	37
Q0	0.0897 ± 0.01	-10.0 ± 0.06	0.949 ± 0.06	Phosphate saline	20
Q1	0.198 ± 0.006	-9,51 ± 0.02	0.860 ± 0.03		
Q2	2.74 ± 0.2	-7.89 ± 0.04	1.00 ± 0.05		

Supplementary Table 3. ECG parameters recorded for heart toxicity assessment for different injected formulations.

	Doxo		Doxo + D0-PS		Doxo + D0-PS-C16		D0-PS		D0-PS-C16	
Day	0	14	0	14	0	14	0	14	0	14
HR (BPM)	496 ± 40	505 ± 48	466 ± 50	559 ± 26	450 ± 20	533 ± 34	485 ± 80	530 ± 27	553 ±60	558 ± 32
RR interval (ms)	122 ± 9	120 ± 12	130 ± 10	108 ± 5	134 ± 6	113 ± 7	126 ± 20	113 ± 6	110 ± 10	109 ± 7
PR interval (ms)	33.9 ± 4	36.1 ± 5	35.1 ± 2	38.2 ± 3	36.5 ± 5	37.7 ± 5	34.8 ± 1	36.3 ± 5	35.0 ± 3	39.0 ± 5
PR segment (ms)	22.3 ± 3	24.7 ± 3	22.8 ± 3	27.1 ± 4	25.6 ± 6	26.1 ± 6	22.6 ± 2	24.8 ± 5	23.0 ± 3	27.2 ± 5
P wave (ms)	11.6 ± 2	11.4 ± 2	12.3 ± 2	11.1 ± 2	10.9 ± 2	11.5 ± 2	12.2 ± 2	11.5 ± 2	11.9 ± 2	11.8 ± 1
P amplitude (mV)	0.062 ± 0.03	0.064 ± 0.02	0.098 ± 0.02	0.074 ± 0.01	0.066 ± 0.02	0.059 ± 0.01	0.095 ± 0.02	0.061 ± 0.02	0.092 ± 0.04	0.070 ± 0.02
QRS interval (ms)	8.07 ± 0.7	8.56 1±	9.57 ± 1	10.5 ± 1	8.83 ± 2	9.39 ± 1	9.55 ± 1	9.34 ± 1	9.34 ± 0.8	9.44 ± 1
QT interval (ms)	46.3 ± 4	46.4 ±4	46.3 ± 6	41.3 ± 2	47.0 ± 4	44.8 ± 2	48.9 ± 4	42.0 ± 5	47.2 ± 5	43.9 ± 4
PRc interval (ms)	30.8 ± 4	33.2 ± 5	30.8 ± 1	36.9 ± 3	31.5 ± 4	35.5 ± 4	31.2 ± 3	34.1 ± 4	33.5 ± 3	37.3 ± 5
QRSc interval (ms)	7.34 ± 0.9	7.85 ± 1	8.42 ± 1	10.14 ± 1	7.65 ± 1	8.84 ± 1	8.56 ± 1	8.78 ± 1	8.95 ± 1	9.03 ± 1
QTc interval (ms)	41.8 ± 4	42.4 ± 4	40.9 ± 3	39.7 ± 1	40.5 ± 3	41.9 ± 2	41.7 ± 2	39.6 ± 5	43.9 ± 4	41.3 ± 3

Supplementary Table 4. Blood biomarkers measured for toxicity assessment two weeks after formulations injection.

Formulation	Troponin (ng/mL)	NT-pro BNP (ng/L)	Urea (µM)	Creatinine (µM)	Bilirubin total (µM)	Bilirubin conjugated (µM)	AST (U/L)	ALT (U/L)	GGT (U/L)
F1 (Doxo)	3.2 ± 1	< 5	6.3 ± 0.6	24 ± 1	< 2	< 2	69 ± 10	26 ± 2	< 4
F2 (Doxo + D0-PS)	2.9 ± 2	< 5	5.9 ± 0.8	25 ± 1	< 2	< 2	114 ± 30	69 ± 30	< 4
F3 (Doxo + D0-PS-C16)	3.8 ± 3	< 5	6.6 ± 0.8	25 ± 0.5	< 2	< 2	127 ± 60	38 ± 10	< 4
F4 (D0-L-thioate)	1.8 ± 1	ND	7.5 ± 1	25 ± 1	< 2	< 2	89 ± 20	59 ± 20	< 4
F5 (D0- L-thioate - C16)	1.6 ± 1	ND	7.1 ± 0.2	26 ± 1	< 2	< 2	81 ± 20	50 ± 20	< 4
Saline	2.0 ± 0.8	ND	8.4 ± 0.7	26 ± 1	< 2	< 2	79 ± 20	28 ± 2	< 4

Supplementary References

1. K. N. Carter Jr, D. M. Scott, J. K. Salmon, G. S. Zarcone, Confidence limits for the abscissa of intersection of two least-squares lines such as linear segmented titration curves. *Analytical chemistry* **63**, 1270-1278 (1991).
2. D. T. Holmes, K. A. Buhr, Error propagation in calculated ratios. *Clinical Biochemistry* **40**, 728-734 (2007).
