# Supplementary material for: Programmable self-regulated molecular buffers for precise sustained drug delivery
Source: Nat Commun. 2022 Nov 2;13:6504. doi: 10.1038/s41467-022-33491-7 (PMC9630261; doi:10.1038/s41467-022-33491-7)
Supplement: Supplementary file 3 — Reporting Summary [file 41467_2022_33491_MOESM3_ESM.pdf]

nature portfolio

Corresponding author(s): Alexis Vallée-Bélisle

Last updated by author(s): Jul 21, 2022

Reporting Summary

Nature Portfolio wishes to improve the reproducibility of the work that we publish. This form provides structure for consistency and transparency in reporting. For further information on Nature Portfolio policies, see our Editorial Policies and the Editorial Policy Checklist.

Statistics

For all statistical analyses, confirm that the following items are present in the figure legend, table legend, main text, or Methods section.

n/a	Confirmed	
☐	☒	The exact sample size (*n*) for each experimental group/condition, given as a discrete number and unit of measurement
☐	☒	A statement on whether measurements were taken from distinct samples or whether the same sample was measured repeatedly
☒	☐	The statistical test(s) used AND whether they are one- or two-sided<br>*Only common tests should be described solely by name; describe more complex techniques in the Methods section.*
☒	☐	A description of all covariates tested
☒	☐	A description of any assumptions or corrections, such as tests of normality and adjustment for multiple comparisons
☐	☒	A full description of the statistical parameters including central tendency (e.g. means) or other basic estimates (e.g. regression coefficient) AND variation (e.g. standard deviation) or associated estimates of uncertainty (e.g. confidence intervals)
☒	☐	For null hypothesis testing, the test statistic (e.g. F, t, r) with confidence intervals, effect sizes, degrees of freedom and P value noted<br>*Give P values as exact values whenever suitable.*
☒	☐	For Bayesian analysis, information on the choice of priors and Markov chain Monte Carlo settings
☒	☐	For hierarchical and complex designs, identification of the appropriate level for tests and full reporting of outcomes
☒	☐	Estimates of effect sizes (e.g. Cohen's *d*, Pearson's *r*), indicating how they were calculated

Our web collection on statistics for biologists contains articles on many of the points above.

Software and code

Policy information about availability of computer code

Data collection	- Cary WinFLR Scan Software Version: 1.2(147) (Agilent, Santa-Clara, CA USA)<br>- Cary WinFLR Kinetics Software Version: 1.2(146) (Agilent, Santa-Clara, CA USA)<br>- SoftMax® Pro GxP v7 (Molecular Devices, San Jose CA)<br>- Zen 2.3 blue edition (Carl Zeiss Microscopy GmbH, Jena, Germany)<br>- BD FACStationTM 6.1 Software (BD Biosciences, NJ USA)<br>- Chem Station software vC.01.06(94) (Agilent, Santa-Clara, CA USA)<br>- Living Image software v4.7.3 (PerkinElmer, Waltham MA)<br>- LabChart Reader v8.1.9 (ADInstruments, Sydney, Australia)<br>- Chirascan v.4.2.15 (Applied Photophysics, Leatherhead UK)<br>- ImageLab 6.0.0 build 25 (Bio-Rad laboratories, Saint-Laurent, Qc Canada)
Data analysis	- FlowPy 5.2 (Department of Biosciences and Bioengineering, Indian Institute of Technology Guwahati, India)<br>- ImageJ 1.51n (Wayne Rasband, NIH, USA)<br>- Kaleida Graph 4.0 (Synergy software PA, USA)

For manuscripts utilizing custom algorithms or software that are central to the research but not yet described in published literature, software must be made available to editors and reviewers. We strongly encourage code deposition in a community repository (e.g. GitHub). See the Nature Portfolio guidelines for submitting code & software for further information.

Data

Policy information about availability of data

All manuscripts must include a data availability statement. This statement should provide the following information, where applicable:

- Accession codes, unique identifiers, or web links for publicly available datasets
- A description of any restrictions on data availability
- For clinical datasets or third party data, please ensure that the statement adheres to our policy

All data have been deposited on figshare (https://doi.org/10.6084/m9.figshare.20326638).

Human research participants

Policy information about studies involving human research participants and Sex and Gender in Research.

Reporting on sex and gender	*Use the terms sex (biological attribute) and gender (shaped by social and cultural circumstances) carefully in order to avoid confusing both terms. Indicate if findings apply to only one sex or gender; describe whether sex and gender were considered in study design whether sex and/or gender was determined based on self-reporting or assigned and methods used. Provide in the source data disaggregated sex and gender data where this information has been collected, and consent has been obtained for sharing of individual-level data; provide overall numbers in this Reporting Summary. Please state if this information has not been collected. Report sex- and gender-based analyses where performed, justify reasons for lack of sex- and gender-based analysis.*
Population characteristics	*Describe the covariate-relevant population characteristics of the human research participants (e.g. age, genotypic information, past and current diagnosis and treatment categories). If you filled out the behavioural & social sciences study design questions and have nothing to add here, write "See above."*
Recruitment	*Describe how participants were recruited. Outline any potential self-selection bias or other biases that may be present and how these are likely to impact results.*
Ethics oversight	*Identify the organization(s) that approved the study protocol.*

Note that full information on the approval of the study protocol must also be provided in the manuscript.

Field-specific reporting

Please select the one below that is the best fit for your research. If you are not sure, read the appropriate sections before making your selection.

☒ Life sciences ☐ Behavioural & social sciences ☐ Ecological, evolutionary & environmental sciences

For a reference copy of the document with all sections, see nature.com/documents/nr-reporting-summary-flat.pdf

Life sciences study design

All studies must disclose on these points even when the disclosure is negative.

Sample size	We chose our sample sizes to be able to determine significance for all experimental conditions tested and to get reproducible significant statistical difference between experimental conditions. When comparing two groups with pharmacokinetics (e.g. mice injected with PBS vs doxorubicin), 6 animals per conditions corresponds to 80% power at a level of significance of 0.05 in order to detect a difference of 2.0 standard deviations with a t-test. We aim to keep a balance between statistical power, research cost and ethical criteria in choosing the sample size.
Data exclusions	No data were excluded from the analysis.
Replication	Mice studies were conducted once at n=6.
Randomization	For mice studies, animals were randomly allocated into an experimental group at the beginning of the experimentation.
Blinding	Researchers and personnel involved in drug administration were not blinded. Injection volumes, blood collection volume and time-point were standardized in order to prevent bias. Sample were processed and analyzed on a HPLC with a standardized method in order to prevent bias.

Reporting for specific materials, systems and methods

We require information from authors about some types of materials, experimental systems and methods used in many studies. Here, indicate whether each material, system or method listed is relevant to your study. If you are not sure if a list item applies to your research, read the appropriate section before selecting a response.

Materials & experimental systems

n/a	Involved in the study
☒	☐ Antibodies
☐	☒ Eukaryotic cell lines
☒	☐ Palaeontology and archaeology
☐	☒ Animals and other organisms
☒	☐ Clinical data
☒	☐ Dual use research of concern

Methods

n/a	Involved in the study
☒	☐ ChIP-seq
☐	☒ Flow cytometry
☒	☐ MRI-based neuroimaging

Eukaryotic cell lines

Policy information about cell lines and Sex and Gender in Research

Cell line source(s): HeLa and HCT116 cell lines were obtained from ATCC (Manassas, USA).

Authentication: Both cell lines were authentified based on morphology and PCR assays with human-specific primers.

Mycoplasma contamination: Negative for mycoplasma.

Commonly misidentified lines (See ICLAC register): No commonly misidentified cell lines were used in this research work.

Animals and other research organisms

Policy information about studies involving animals; ARRIVE guidelines recommended for reporting animal research, and Sex and Gender in Research

Laboratory animals: 8-weeks-old female CD-1 mice from Charles River.

Wild animals: No wild animals involved.

Reporting on sex: *Indicate if findings apply to only one sex; describe whether sex was considered in study design, methods used for assigning sex. Provide data disaggregated for sex where this information has been collected in the source data as appropriate; provide overall numbers in this Reporting Summary. Please state if this information has not been collected. Report sex-based analyses where performed, justify reasons for lack of sex-based analysis.*

Field-collected samples: No filed-collected samples.

Ethics oversight: Comité de déontologie de l'expérimentation sur les animaux (CDEA), Université de Montréal

Note that full information on the approval of the study protocol must also be provided in the manuscript.

Flow Cytometry

Plots

Confirm that:

☒ The axis labels state the marker and fluorochrome used (e.g. CD4-FITC).

☒ The axis scales are clearly visible. Include numbers along axes only for bottom left plot of group (a 'group' is an analysis of identical markers).

☒ All plots are contour plots with outliers or pseudocolor plots.

☒ A numerical value for number of cells or percentage (with statistics) is provided.

Methodology

Sample preparation: Cells were seeded in a 24-well plate at a density of 20x10^3 cells per well and incubated for 24h. After seeding, culture mediujm was removed, and fresh medium with various concentrations of doxorubicin-aptamer or fluorescent buffer (d0-FAM) was added (2 wells per condition). Cells were incubted 1h at 37oC and rinsed with DPBS 1X. Cells were trypsinized and suspended in a FACS buffer (DPBS with 1% FBS and 0.1% sodium azide (Sigma Aldrich, Oakville ON)).

Instrument: FACS analysis was performed on a FACScalibur (BD Sciences Franklin Lakes NJ).

Software	- Data collection: BD FACStationTM 6.1 Software (BD Biosciences, NJ USA)<br>- Data analysis: FlowPy 5.2 (Department of Biosciences and Bioengineering, Indian Institute of Technology Guwahati, India)
Cell population abundance	Fluorescence measurements were recorded with a linear scaling on a FL1-A fluorescence channel for fluorescein or an FL2-A channel for doxorubicin. Each measurement consisted of 20,000 events and was repeated 3 times.
Gating strategy	All events were first gated on the FSC-A axis vs. SSC-A, then on the SSC-W axis vs SSC-H and finally FSC-W vs FSC-H to remove dead cells in the 3 dimensions. Finally, fluorescence on FL1-A axis (fluorescein) or FL1-B (doxorubicin) was measured on the gated population and reported as the median fluorescence normalized on cell counts for each sample.

☒ Tick this box to confirm that a figure exemplifying the gating strategy is provided in the Supplementary Information.
